# Identification of a prognostic signature based on ammonia metabolism-related genes in clear cell renal cell carcinoma: an integrated analysis of bulk and single-cell transcriptomics

**DOI:** 10.3389/fimmu.2026.1765098

**Published:** 2026-04-23

**Authors:** Yao Jiang, Tian Zhang, Jie Fan, Yansheng Su, Shaoyi Qiao, Jintao Ji, Xiangnan Hu, Shuchang Zhou, Yingjuan Wei, Lina Du, Bo Yang, Wuhe Zhang

**Affiliations:** 1 Department of Urology, Air Force 986 Hospital, Xi’an, Shaanxi, China; 2 Department of Urology, Xijing Hospital of Air Force Military Medical University, Xi’an, Shaanxi, China

**Keywords:** ammonia induced cell death, clear cell renal cell carcinoma, metabolic reprogramming, prognostic genes, single-cell sequencing analysis

## Abstract

**Background:**

Clear cell renal cell carcinoma (ccRCC) is an angiogenic tumor originating from proximal tubule epithelial cells. Ammonia induced cell death is closely associated with carcinogenesis, but its potential mechanism in ccRCC remains unclear and requires further investigation.

**Methods:**

The transcriptomic data of ccRCC and the genes related to ammonia induced cell death were retrieved from public resources. Candidate genes were ascertained by taking the common part of the Differentially Expressed Genes (DEGs) and the Ammonia death-related genes (ADRGs). Prognostic genes were filtered using machine learning and a prognostic model was established. ccRCC patients were segmented into a high-risk group (HRG) and a low-risk group (LRG) in accordance with the risk score values. Functional enrichment, immune infiltration analysis, somatic mutation and Reverse transcription quantitative PCR (RT-qPCR) were also executed. Key cells were identified at the single-cell level, and analyses including cell communication and pseudo-temporal analysis were conducted.

**Results:**

The risk model was constructed from 6 prognostic genes (RGS20, ADA, AICDA, SLC12A5, RUFY4 and CDK5RAP3). RGS20, ADA, AICDA, SLC12A5, RUFY4 and CDK5RAP3 were significantly upregulated in ccRCC group, and the RT - qPCR analysis results were consistent (p < 0.05). Enrichment analysis suggested that HRG and LRG were linked to pathways, such as “olfactory transduction” signaling pathway (p < 0.05). Sixteen immune cells with differential abundance between HRG and LRG were identified, such as activated B cells. The most frequent type of mutation between HRG and LRG was missense mutation. Malignant cells (MCs) were identified as the key cells. Moreover, frequent interactions between MCs and T cells were observed in ccRCC, and 6 prognostic genes were involved in the regulation of MCs’ expression.

**Conclusion:**

This study identified 6 prognostic genes for ccRCC, MCs were identified as the key cells. We explored potential mechanisms and prognostic associations in ccRCC, providing new insights for potential therapeutic strategies.

## Introduction

Clear cell renal cell carcinoma (ccRCC) originates from the epithelial cells of the proximal renal tubules and is the most common subtype of renal cancer, accounting for approximately 70%-80% of all renal cancer case (1). This disease exhibits not only high tumor heterogeneity, a high metastasis rate, and poor prognosis in advanced stages (2), but also distinct metabolic abnormalities, specifically manifested as altered lipid and glycogen storage patterns (3). Notably, the disease often remains asymptomatic until advanced stages (III or IV), leading to delayed diagnosis and dismal survival outcomes (4). These challenges underscore the urgent need to identify novel therapeutic targets and optimize treatment strategies for ccRCC.

Ammonia-induced cell death (ammonitosis) is a recently described form of regulated cell death triggered by excessive intracellular ammonia accumulation. This process disrupts lysosomal and mitochondrial function, primarily through dysregulated glutamine metabolism (5). Under physiological conditions, ammonitosis plays a critical role in immune homeostasis by eliminating effector CD8+ T cells post-infection to prevent autoimmune responses (6). Emerging evidence links aberrant ammonia metabolism to various pathologies, including cancer, liver diseases, and neurodegenerative disorders (7, 8). However, the mechanistic role of ammonitosis in ccRCC pathogenesis remains poorly understood. Given the metabolic reprogramming inherent to ccRCC, particularly its reliance on glutamine metabolism, investigating ammonia-induced cell death could unveil novel pathways driving tumor progression and therapy resistance.

Single cell RNA sequencing (scRNA seq) technology has become a powerful tool for studying cell biology and is widely used in basic and clinical research. It can characterize cellular heterogeneity, identify rare but important cell types, and also explore communication networks and interactions between cells (9). In ccRCC, scRNA-seq has identified malignant cell subpopulations, immune microenvironment interactions, and lineage-specific transcriptional programs that conventional bulk sequencing cannot resolve (10). Integrating scRNA-seq with functional studies offers a powerful approach to elucidate how ammonia metabolism influences key cellular players in ccRCC progression.

This study utilized transcriptomic data from ccRCC and control samples in public databases to perform differential expression analysis. Through bioinformatics methods, we further identified prognostic genes playing crucial roles in ccRCC and associated with ammonia metabolism. We then conducted an in-depth analysis of the potential mechanisms by which these prognostic genes function in ccRCC. Subsequently, single-cell analysis was performed to identify key cells, and pseudo-time analysis was conducted to explore the dynamic expression of prognostic genes during the differentiation processes of these key cells. Finally, RT-qPCR validation was performed to elucidate the prognostic significance of ammonia metabolism in ccRCC, aiming to provide a theoretical basis for optimizing treatment strategies and advancing personalized therapy for ccRCC.

## 2 Materials and methods

### 2.1 Data collection

The transcriptomic, mutation, clinical, and survival data for ccRCC were retrieved from TCGA database (https://www.cancer.gov/ccg/research/genome-sequencing/tcga, downloaded on April 17th, 2025). Specifically, the TCGA-KIRC dataset included 538 ccRCC kidney tissue samples along with 72 paracancerous tissue samples, and 534 tumor kidney tissue samples with survival data. The `createFolds` function from the caret package was used to split the samples with survival information in the TCGA-KIRC dataset into 10 folds. A random seed of 512000 was set to ensure reproducibility of the results. The first 7 folds (70%) were used as the training set 1 (374 samples), and the remaining 3 folds (30%) were designated as validation set 1 (160 samples). The E-MTAB-1980 dataset containing transcriptome data of ccRCC and survival information was Retrieved from the ICGC database (https://icgc.org/content/icgc-home-0) as validation set 2. It included 101 ccRCC renal tissue samples with survival information and was downloaded on April 17, 2025. scRNA-seq dataset related to ccRCC was obtained from the GEO database. Specifically, the GSE159115 dataset (platform: GPL16791) included 7 ccRCC patient kidney tissue samples and 6 paracancerous samples. A total of 467 ADRGs were obtained from the literature (6) (**Supplementary Table 1**).

### 2.2 Differential expression analysis

The DEGs between ccRCC (Tumor) and normal samples in TCGA-KIRC were discovered using the R package “DESeq2” (v 3.54.0) (11). The filtering conditions were adjusted p - value < 0.01 and |log_2_ fold change (FC)| > 2. The top 10 up- and down-regulated DEGs ranked by log_2_FC value from high to low were marked on the volcano plot using R package “ggplot2” (v 3.5.1) (12). The expression trends DEGs were visualized in the heatmap using R package “ComplexHeatmap” (v 2.18.0) (13).

### 2.3 Identification and analysis of candidate genes

To identify candidate genes, the R package “ggvenn” (v 0.1.10) (14) was used to find the intersection of DEGs and ADRGs to obtain candidate genes. KEGG and GO analyses were executed for candidate genes using the R package “clusterProfiler” (v 4.10.1) (15) (adjusted p < 0.05, corrections were carried out using the Benjamini-Hochberg (BH) method) to investigate the functions of candidate genes. Additionally, the candidate genes were evaluated by protein - protein interaction (PPI) network using the Search Tool for the Retrieval of Interacting Genes (STRING) database (https://string-db.org/) (confidence > 0.4). Subsequently, the candidate genes were visualized by means of Cytoscape software (v 3.10.2) (16).

### 2.4 Identification and analysis of prognostic genes

To further screen for prognostic genes, based on ccRCC samples with survival information in the training set 1, a univariate Cox regression analysis (p < 0.001, Hazard Ratio (HR) ≠ 1) was executed on the candidate genes using the R package “survival” (v 3.7-0) (17). Additionally, the PH assumption test (p > 0.05) was executed by means of the R package “survival” (v 3.7-0) (18) to obtain genes with prognostic value. Subsequently, based on the genes with prognostic value, LASSO regression analysis was executed by means of the R package “glmnet” (v 4.1.8) (18) (family =“cox”, nfolds = 5). After 10 - fold cross - validation, the model corresponding to the lambda.min value was selected as the optimal model to screen out the prognostic genes.

### 2.5 Construction of risk model

The prognostic genes were used for constructing the risk model with the following formula: Riskscore = ∑iCoefficient(i)*Expression of gene(i). In the training set 1, ccRCC samples with survival information were divided into a HRG and a LRG in accordance with the median risk score. The Kaplan-Meier (K-M) curves and risk curve of the HRG and LRG were plotted, and the prognostic ability of the risk model was evaluated using the R package “survminer” (v 0.4.9) (18). Furthermore, the model’s predictive performance of 3, 5, 7 years survival rates was evaluated using ROC. 1 > AUC values > 0.6, created with the R package “survivalROC” (v 1.0.3.1) (19). Then, to validate the risk model, the same analytical approach served to assess the model in ccRCC samples from validation set 1 and validation set 2.

### 2.6 Correlation analysis of clinical characteristics, human protein atlas, and expression analysis of prognostic genes

To explore the differences in risk scores among different clinical characteristic subgroups (including Age, Stage, Gender, T stage, M stage, N stage), based on the ccRCC samples from the training set 1, the disparities in risk scores among clinical characteristic subgroups were evaluated by means of the Wilcoxon test (p < 0.05). To explore the protein manifestation of prognostic genes in ccRCC tissue samples and paracancerous samples, the protein manifestation of prognostic genes was analyzed utilizing the HPA database. Subsequently, to profile the expression manifestation of prognostic genes in tumor and normal samples, the manifestation levels of prognostic genes were compared through the Wilcoxon test (p < 0.05).

### 2.7 GSEA and GSVA

To further elucidate the signaling pathways associated with the HRG and LRG in the training set 1, the DEGs between the HRG and LRG were detected through the R package “DESeq2” (v 3.54.0) (11), and calculated the log_2_FC. The “c2.KEGG.v2022.1.Hs.symbols” was retrieved as the background set from the MSigDB, and GSEA of the DEGs was performed using the R package “clusterProfiler” (v 4.10.1) (15) with adjust p < 0.05 (corrections were carried out using the BH method), and |(NES)| > 1. Then, to analyze the disparities in the biological functions and signaling pathways of genes between the HRG (n = 141) and LRG (n = 233), based on the ccRCC samples with survival information in the training set 1, the “c2.cp.kegg.v2023.1.Hs.symbols.gmt” was downloaded from MSigDB to serve as the background. GSVA scores for each sample were calculated using the R package “GSVA” (v 1.50.0) (20). The R package “limma” (v 3.56.2) (21) was then applied to compare functional enrichment pathway differences between HRG and LRG, with the thresholds set at |t| > 2 and p < 0.05.

### 2.8 Immune checkpoints and immune factors analysis

In order to understand the associations between immune checkpoints, immune factors and the occurrence of ccRCC, 65 immune checkpoints were obtained from the literature (22). Then, based on the ccRCC samples in training set 1, a correlation analysis between the prognostic genes and immune checkpoints was conducted using the R package “psych” (v 2.4.3) (23) (|(cor)| > 0.30, p < 0.05). After that, in order to explore the correlation between the prognostic genes and immune factors, different immune factor sets (including immune inhibitors, immune stimulants, chemokines, receptors, major histocompatibility complex (MHC)) were collected from TISIDB. Based on the ccRCC samples in the training set 1, a correlation analysis between the prognostic genes and these immune factors was conducted using the R package “psych” (v 2.4.3) (|cor| > 0.30, p < 0.05).

### 2.9 Immunotherapy analysis

To evaluate the infiltration status of stromal cells and immune cells in ccRCC, the stromal score, immune score, and ESTIMATE score, tumor purity of ccRCC samples were calculated using the ESTIMATE algorithm, and the differences in each score between the HRG and LRG were compared through the Wilcoxon test (p < 0.05).

### 2.10 Immune infiltration analysis

To investigate immune cell infiltration in HRG and LRG from the training set 1, the ssGSEA algorithm from the R package “GSVA” (v 1.50.0) (20) served to calculate infiltration scores for 28 immune cells (MMC3.GMT immune gene set) (51) across both HRG and LRG. The Wilcoxon test served to evaluate discrepancies in infiltration scores between HRG and LRG. Immune cell types with significant differential infiltration (adjust p < 0.05, corrections were carried out using the BH method) were selected. Then, correlation analysis was executed using the R package “psych” (v 2.4.3) to explore relationships among differentially infiltrated immune cell types and between these immune cells and prognostic genes, with thresholds set at |cor| > 0.30 and p < 0.05.

### 2.11 Analysis of tumor mutation and drug sensitivity

To explore the somatic mutation status between the HRG and LRG in ccRCC patients in the training set 1, we retrieved somatic mutation data (in MAF format) of ccRCC directly from TCGA database using the tcga_load(study = "KIRC") function in the TCGAmutations package. TCGAmutations packages are typically based on the standardized processing version of the TCGA Firehose, containing somatic mutations identified through a unified workflow to ensure data comparability and reliability. Based on the HRG and LRG in the ccRCC samples of the training set 1, the gene mutation status of each ccRCC sample was characterized by means of R package “Maftools” (v 2.18.0) (24), and the top 20 common mutant genes in the HRG and LRG were presented. Generally, a higher half-maximal inhibitory concentration (IC_50_) value indicates lower drug sensitivity. To evaluate the drug sensitivity profiles of ccRCC patients in training set 1, IC_50_ values for 138 conventional drugs from the GDSC database were calculated using the R package “pRRophetic” (v 0.5) (25). Wilcoxon test was then utilized for identify notable discrepancies in drug sensitivity between HRG and LRG from ccRCC samples in the training set 1 (p < 0.05).

### 2.12 Data quality control and cell annotation

The GSE159115 dataset was evaluated with the aid of the R package “Seurat” (v 5.1.0) (21). The following screening guidelines were adopted: Cells containing fewer than 200 genes were excluded from the analysis. Moreover, cells had to meet the following criteria: 200 < nFeature_RNA (genes per cell) < 6000, 200< nCount_RNA (total RNA count per cell) < 40000, and percentage.mt (proportion of mitochondrial gene expression) < 25%. After QC, low - quality cells were removed. Subsequently, LogNormalize normalization was performed using the NormalizeData function. The FindVariableFeatures function in the R package “Seurat” (v 5.1.0) (21) was utilized to identify highly variable genes (HVGs). The data was normalized utilizing the ScaleData function in the R package “Seurat” (v 5.1.0) (21), and then the batch effect among samples was observed. The HVGs were subjected to Principal Component Analysis (PCA) utilizing the R package “Seurat” (v 5.1.0) (21). In the JackStraw function, the principal components with genes having smaller enrichment p were retained (p < 0.05). The cells in the GSE159115 dataset were classified with the use of the R package “Seurat” (v 5.1.0) (resolution = 0.2). Subsequently, the classified cell clusters were annotated on the basis of the marker genes in the literature (26, 27) and CellMarker2 Website. The R package “Seurat” (v 5.1.0) (21) was used to draw a Uniform Manifold Approximation and Projection (UMAP) plot to display the annotated cell clusters. To understand the distribution differences of the annotated cell clusters between different groups, the R package “ggplot2” (v 3.5.1) (28) was used to plot the distribution proportions of the annotated cell clusters.

### 2.13 Identification of key cells and function analyses, cell-to-cell communication, pseudo-temporal analyses

To understand the expression differences of the annotated cell clusters between the normal and ccRCC groups, the R package “ggplot2” (v 3.5.1) (28) was used to plot the distribution proportions of the annotated cell clusters in the normal and ccRCC groups, the Wilcoxon test was employed to contrast the prognostic genes expression disparity of the annotated cell clusters between the normal and tumor (p < 0.05). The cells that were differentially expressed and had high expression of most prognostic genes were selected as key cells. To delve into the biological pathways and functions related to the key cells and annotated cell clusters, ReactomeGSA (https://github.com/reactome/ReactomeGSA) was utilized to perform functional enrichment analysis on the annotated cell clusters in the GSE159115 dataset (p < 0.05). Additionally, cell-to-cell communication analysis was executed using the R package “CellChat” (v 1.6.1) (29) to investigate interactions between key cell types and other annotated cell types in both the disease and control samples from the GSE159115 dataset. This analysis generated cell-cell communication networks and determined the expression levels of receptors and ligands, highlighting the number and strength of interactions and the intensity of receptors and ligands. Subsequently, the reduceDimension functions of the R package “Monocle” (v 2.30.1) (30) and R package “DDRTree” (v 0.1.5) (31) were used to conduct secondary dimensionality reduction on the key cells (max_components=2, resolution = 0.2) in the GSE159115 dataset. Then, the R package “Monocle” (v 2.30.1) was used to analyze the cell differentiation trajectories and to construct the cell trajectory plot of key cell types. The expression changes of prognostic genes throughout the pseudo-temporal progression were also analyzed.

### 2.14 RT-qPCR

To preliminarily verify the mRNA expression trend of prognostic genes in clinical samples, RT-qPCR experiments were conducted. Specifically, 10 tissue samples (5 ccRCC tumors and 5 normal controls) were derived from the clinic in the department of Urology, Air Force 986th Hospital of Air Force Medical University. All participants were given informed consent. This study has been approved by the Ethics Committee of the Air Force 986th Hospital (approval number: KYLL2025-986-177). All primers were listed in **Supplementary Table 2**. We used the CFX96 Connect Real - time Quantitative Fluorescence PCR Instrument (Bio - Rad, USA) to perform the qPCR assay. The conditions for thermal cycling were as follows: First, there was a pre - denaturation step. During this step, the temperature was set at 95°C, and it was maintained for 1 minute. After that, a total of 40 cycles were performed. The 2^-ΔΔCT^ method was employed to calculate the relative quantification of mRNAs. Once the RT - qPCR results were generated, they were first exported to Excel and subsequently imported into Graphpad Prism 10 (https://www.graphpad.com/) for statistical analysis and visualization (p < 0.05).

### 2.15 Statistical analysis

Bioinformatics were implemented with the R (v 4.3.3). Wilcoxon test was utilized to evaluate disparities. For qRT-PCR analysis, the T-test was employed for statistical comparisons, and p < 0.05.

## 3 Results

### 3.1 Ascertainment and exploration of functions of 96 candidate genes

**Figure 1:**
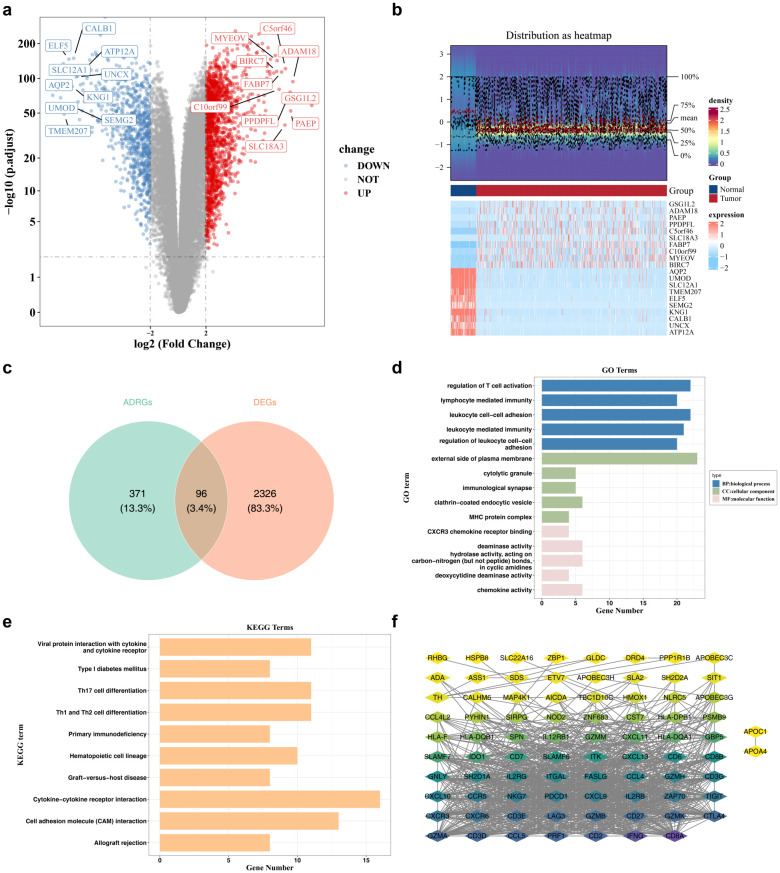
Screening of differentially expressed genes. **(a)** Volcano plot of DEGs, with red representing upregulation and blue representing downregulation. **(b)** Heatmap of DEGs, with red indicating higher expression levels and blue indicating lower expression levels. **(c)** The Venn diagram shows the intersection of DEGs and 467 ADRGs obtained from the literature, resulting in a total of 96 candidate genes. **(d)** Result map of GO enrichment analysis. Blue represents BP, green represents CC, and pink represents MF. **(e)** Result map of KEGG enrichment analysis. **(f)** PPI network diagram. The colors range from yellow, green to purple, representing the degree of connectivity from low to high.

By comprehensively analyzing the DEGs in the TCGA-KIRC, we ascertained a total of 2,422 DEGs, and it was found that there were 1,602 up - regulated genes and 820 down - regulated genes in ccRCC samples (**Figures 1a, b**). The intersection of 2,422 DEGs and 467 ADRGs yielded 96 candidate genes (**Figure 1c**). Subsequently, a total of 517 GO functional annotation results were obtained, including 414 enrichments in BPs like “regulation of T cell activation”, 30 in CCs like “cytolytic granule” and 73 in MFs like “CXCR3 chemokine receptor binding” (adj.p < 0.05) (**Figure 1d****) (****Supplementary Table 3**). Next, 102 KEGG pathways such as “Endocrine resistance” were enriched (p < 0.05) (**Figure 1e****) (****Supplementary Table 4**). Finally, the outcomes of the PPI network disclosed that 15 candidate genes were isolated, and among the remaining 81 candidate genes had interactions (**Figure 1f**).

### 3.2 Construction and of validation a well-performing risk model in 6 prognostic genes

**Figure 2:**
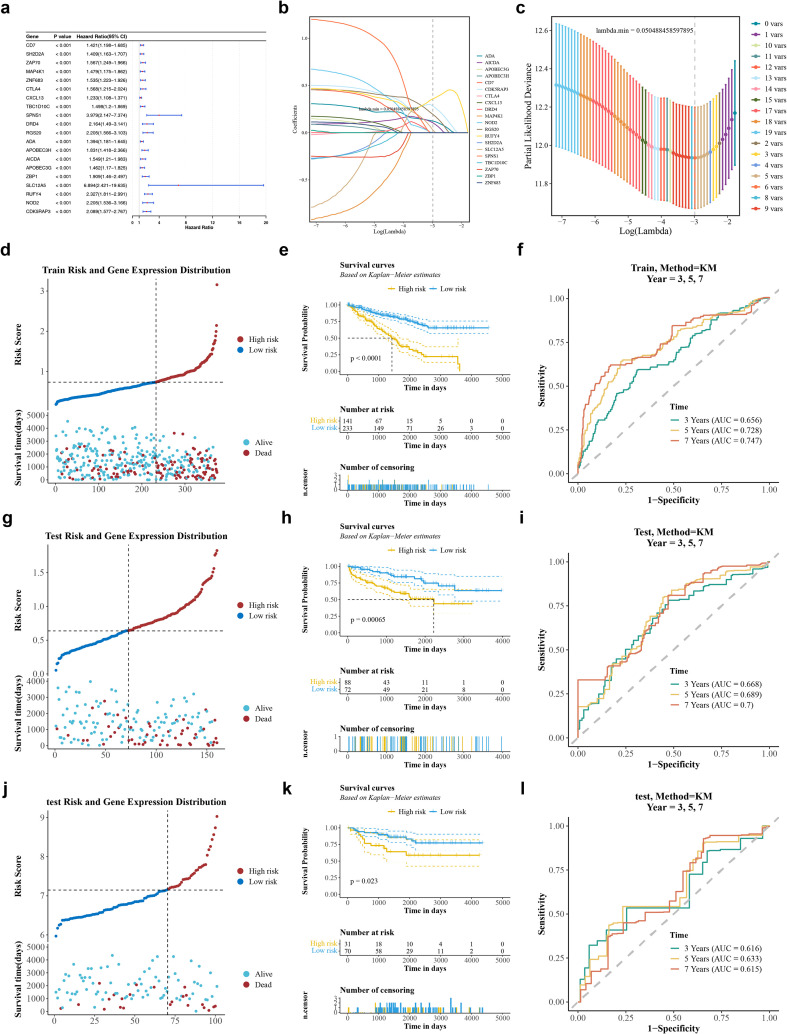
Construction of the risk model. **(a)** Forest plot of univariate Cox analysis. The red dots in the figure represent the HR values, and the blue line segments on both sides represent the 95% confidence interval of the HR values. The longer the line segment, the smaller the research result; the middle line (HR = 1) represents the null line. If the blue line segment crosses this null line, it indicates that there is no difference between the cancer group and the control group. **(b)** Error plot of Lasso cross - validation. **(c)** Plot of Lasso gene coefficients. **(d)** Analysis plot of risk score and survival status in the training set. The red represents the high - risk group, and the blue represents the low - risk group. **(e)** KM survival curve in the training set. The yellow represents the high - risk group, and the blue represents the low - risk group. **(f)** ROC curve of the KM curve in the training set. **(g)** Analysis plot of risk score and survival status in the internal validation set. **(h)** KM survival curve in the internal validation set. **(i)** ROC curve of the KM curve in the internal validation set. **(j)** Analysis plot of risk score and survival status in the external validation set. **(k)** KM survival curve in the external validation set. **(l)** ROC curve of the KM curve in the external validation set. Green, yellow, and orange represent the AUC values at 3 years, 5 years, and 7 years respectively.

Overall, 20 candidate prognostic genes were detected (**Figure 2a**) and the PH assumption test was performed (**Supplementary Table 5**) (p > 0.05). Overall, 6 prognostic genes (RGS20, ADA, AICDA, SLC12A5, RUFY4 and CDK5RAP3) were then selected using LASSO, with a minimum lambda of 0.050488458597895 as the threshold (**Figures 2b, c**). Then, a risk model was developed based on the 6 prognostic genes, successfully stratifying 374 ccRCC patients from the training set 1 into 2 groups: HRG (141 samples) and LRG (233 samples), using a median risk score of 0.7354565. Risk curves and survival status distribution in the training set 1 clearly distinguished the two groups, with HRG patients showing higher mortality rates and shorter survival times (**Figure 2d**). K-M survival further demonstrated notably lower survival probabilities for HRG patients (p < 0.0001) (**Figure 2e**). ROC curves indicated strong predictive performance, with all AUC values exceeding 0.6 in the training set 1, and the AUC values of the 3-, 5-, and 7-year survival rates were 0.656, 0.728, and 0.747, respectively (**Figure 2f**). Similarly, 160 ccRCC patients from the validation set 1 were classified into HRG (88 samples) and LRG (72 samples) using the same median risk score of 0.6410694. In the risk curves and survival status, the HRG had a high mortality rate (**Figure 2g**). K-M survival curves further demonstrated notably lower survival probabilities for HRG patients (p = 0.00065) (**Figure 2h**), and the AUC values of the 3-, 5-, and 7-year survival rates were 0.668, 0.689, and 0.7, respectively (**Figure 2i**). Finally, these results were successfully validated in the validation set 2 (**Figures 2j–l**). These findings suggest that the developed risk model has the potential to stratify ccRCC patients based on their prognostic profiles, though its clinical utility requires further validation against established prognostic factors.

### 3.3 Clinical features, transcript abundance of prognostic genes

**Figure 3:**
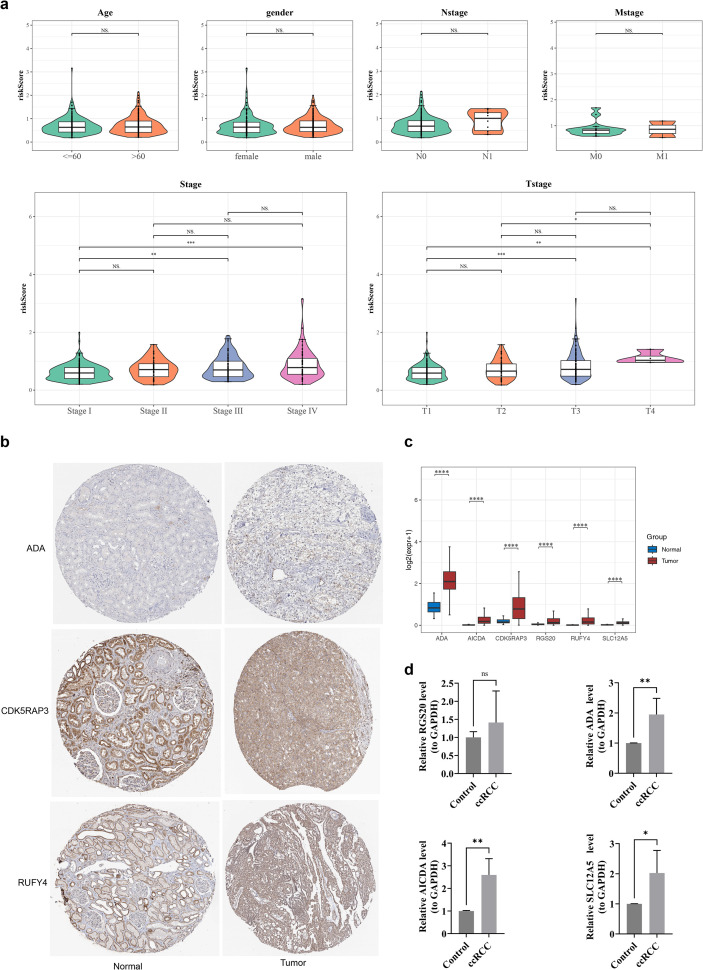
Clinicopathological analysis of prognostic features. **(a)** Differential analysis of risk scores for clinical feature subtypes Age, Gender, Nstage, Mstage, Stage, and Tstage. ns represents insignificant, *p<0.05, **p<0.01, ***p<0.001, ****p<0.0001. **(b)** IHC of ADA, CDK5RAP3, and RUFY4 in the normal group and the ccRCC tissue group. **(c)** Box plot of the expression levels of prognostic genes. *p<0.05, **p<0.01, ***p<0.001, ****p<0.0001. **(d)** Results of RT-qPCR analysis. ns represents insignificant, *p<0.05, **p<0.01.

Through a comprehensive series of analyses, results illustrated that differences in risk score manifestation were found among the subgroups of T stage and Stage (p < 0.05), and the expression levels of risk scores were higher in the Stage IV and T4 Stage subgroup (**Figure 3a**). The HPA results denoted that the manifestation levels of the corresponding proteins of ADA, CDK5RAP3, and RUFY4 were detected to be drastically higher in ccRCC tissues (**Figure 3b**). In addition, the expression of prognostic genes showed significant differences between ccRCC and control samples. Among them, RGS20, ADA, AICDA, SLC12A5, RUFY4 and CDK5RAP3 were significantly upregulated in ccRCC group (p < 0.05) (**Figure 3c**). Similarly, in the RT-qPCR analysis, ADA, AICDA, SLC12A5, RUFY4 and CDK5RAP3 were notably upregulated in the ccRCC group, RGS20 expression trended upward, but no biologically relevant (p < 0.05) (**Figure 3d**).

### 3.4 Signaling pathways enrichment analysis in HRG and LRG patients of ccRCC

**Figure 4:**
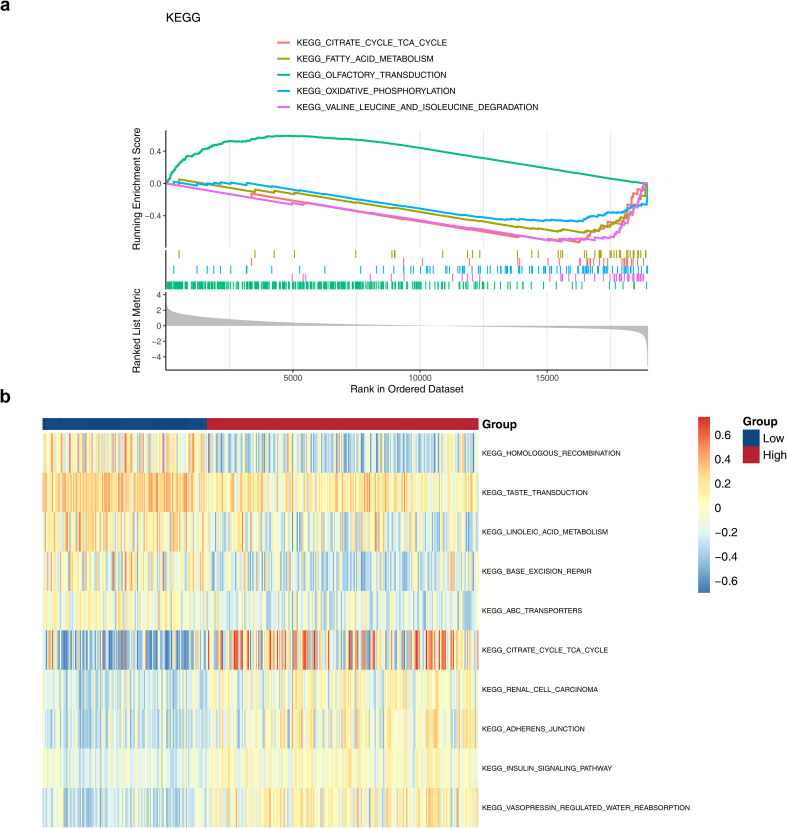
GSEA and GSVA analyses of the high- and low-risk groups. **(a)** GSEA-KEGG enrichment analysis of the high- and low-risk groups. The curves in the figure represent different pathways. The horizontal axis represents the ranking of genes in the sorted dataset, and the vertical axis represents the running enrichment score. The top peaks of the curves show the enrichment degree of each gene set in the dataset. The bars below mark the positions of genes, and the density plot at the bottom represents the overall distribution of these genes in the entire dataset. **(b)** GSVA enrichment analysis of the high- and low-risk groups. Yellow represents upregulation of the gene set in its corresponding group, and blue represents downregulation.

A total of 27 pathways were obtained between the HRG and LRG, such as olfactory transduction, oxidative phosphorylation (**Figure 4a**) (**Supplementary Table 6**) (adjust p < 0.05, |NES| >1). These outcomes demonstrated that these pathways participated in the pathogenesis of ccRCC. In the GSVA analysis, 141 pathways with differential expression were detected between HRG and LRG patients (**Figure 4b**) (**Supplementary Table 7**) (|t| > 2, p < 0.05). Notable up-regulated pathways in HRG included “citrate cycle TCA cycle”, etc. and homologous recombination in HRG included “propanoate metabolism”. These outcomes demonstrated that these pathways participated in the pathogenesis of ccRCC.

### 3.5 The immune functions of the 6 prognostic genes

**Figure 5:**
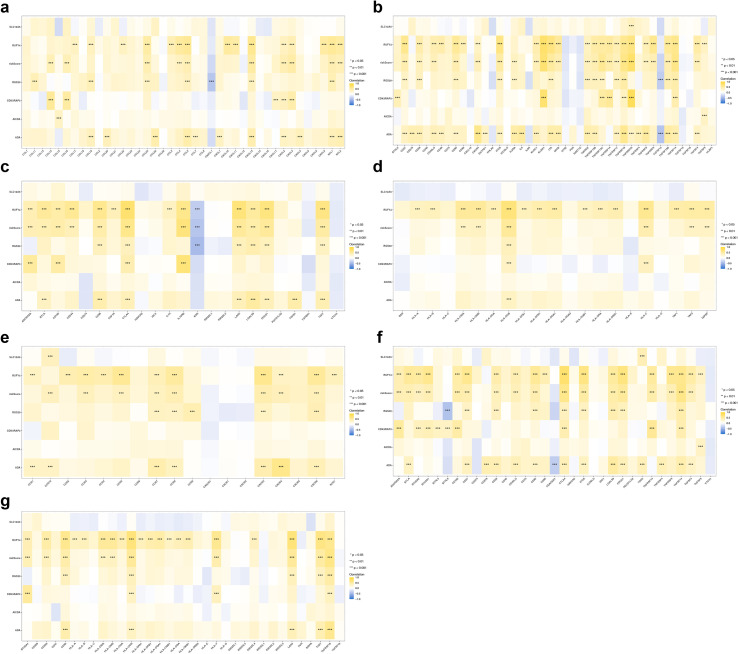
Immune checkpoint analysis. **(a)** Correlation analysis between immune checkpoint genes and risk scores. **(b)** Correlation analysis between prognostic genes and risk scores. Correlation analysis between prognostic genes and risk scores for MHC **(c)**, Immunostimulator **(d)**, Immunoinhibitor **(e)**, chemokine **(f)**, and receptor **(g)** and immune regulators. Yellow represents positive correlation, blue represents negative correlation, *p<0.05, **p<0.01, ***p<0.001, ****p<0.0001.

In the immune factor analysis, RUFY4 showed the highest direct association with the chemokine CXCL13 (cor = 0.57, p < 0.001), and RGS20 showed the highest negative association with the chemokine CX3CL1 (cor = - 0.38, p < 0.001) (**Figure 5a**). CDK5RAP3 showed the highest direct association with the immune stimulant TNFRSF25 (cor = 0.83, p < 0.001), and ADA showed the highest negative association with the immune stimulants TNFSF13 (cor = - 0.40, p < 0.001) (**Figure 5b**). RUFY4 showed the highest direct association with the immune inhibitors CTLA4 (cor = 0.74, p < 0.001), and RGS20 showed the highest negative correlation with the immune inhibitors factor KDR (cor = - 0.46, p < 0.001) (**Figure 5c**). RUFY4 showed the highest positive correlation with the MHC HLA-DOB (cor = 0.64, p < 0.001) (**Figure 5d**). ADA showed the highest positive association with the chemokine receptor CXCR4 (cor = 0.60, p < 0.001) (**Figure 5e**). In addition, the immune checkpoint CTLA4 demonstrated the highest direct association with RUFY4 (cor = 0.74, p < 0.001), and RGS20 demonstrated the highest negative correlation with the immune checkpoint BTNL9 (cor = - 0.41, p < 0.001) (**Figure 5f**). Meanwhile, the immune checkpoint LAG3 showed the highest direct association with RUFY4 (cor = 0.68, p < 0.001) (**Figure 5g**).

### 3.6 Immunotherapy and immune microenvironment in ccRCC patients

**Figure 6:**
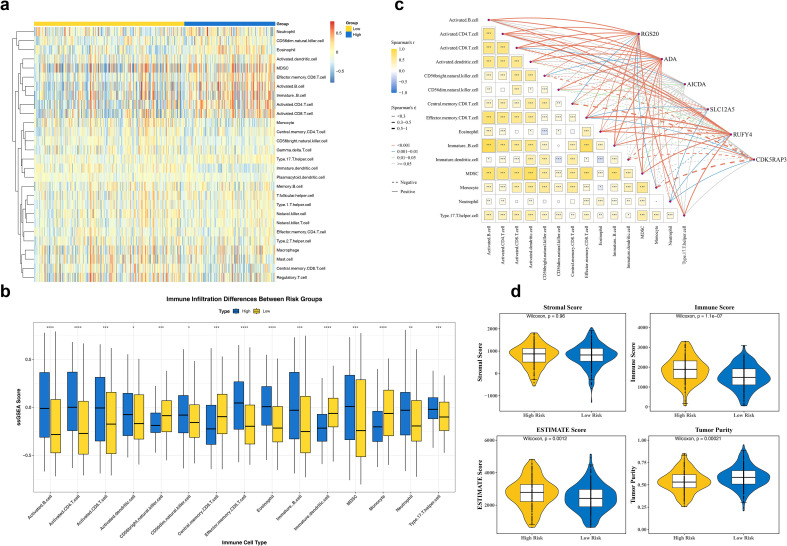
Immune infiltration analysis. **(a)** Heatmap of the content of different cells in ssGSEA. Blue represents the high-risk group, and yellow represents the low-risk group. The redder the color, the higher the cell content. **(b)** Expression of immune infiltrating cells in samples of high- and low-risk groups. **(c)** Correlation analysis between differentially expressed immune cells and prognostic genes. The color changing from yellow to blue indicates the correlation changing from positive to negative. **(d)** Box plot comparing immune scores, stromal scores, estimated scores, and tumor purity in high- and low-risk groups. Yellow represents the high-risk group, and blue represents the low-risk group. Yellow indicates positive correlation, and blue indicates negative correlation. *p<0.05, **p<0.01, ***p<0.001, ****p<0.0001.

Immunity is crucial for the initiation and progression of ccRCC (32), immune infiltration analysis identified that such as activated B cells accounted for the highest proportion in HRG patients (**Figure 6a**) and 15 immune cells with differential abundance between HRG and LRG, such as eosinophils. For instance, eosinophils were higher in HRG (**Figure 6b**) (adjust p < 0.001). Correlation analysis demonstrated that activated B cells were marked positive association with immature B cells (cor = 0.85, p < 0.05), and RUFY4 showed the highest direct association with the effector memory CD8 T cells (cor = 0.52, p < 0.001), CDK5RAP3 illustrated the highest negative correlation with the Monocyte (cor = - 0.50, p < 0.001) (**Figure 6c**). The Immune Score, ESTIMATE Score were all found to be higher in the HRG, and tumor purity were all found to be higher in the LRG (**Figure 6d**). It was indicated that a stronger immune response was present in the samples of the HRG.

### 3.7 Tumor mutation and drug sensitivity in the HRG and LRG of ccRCC patients

**Figure 7:**
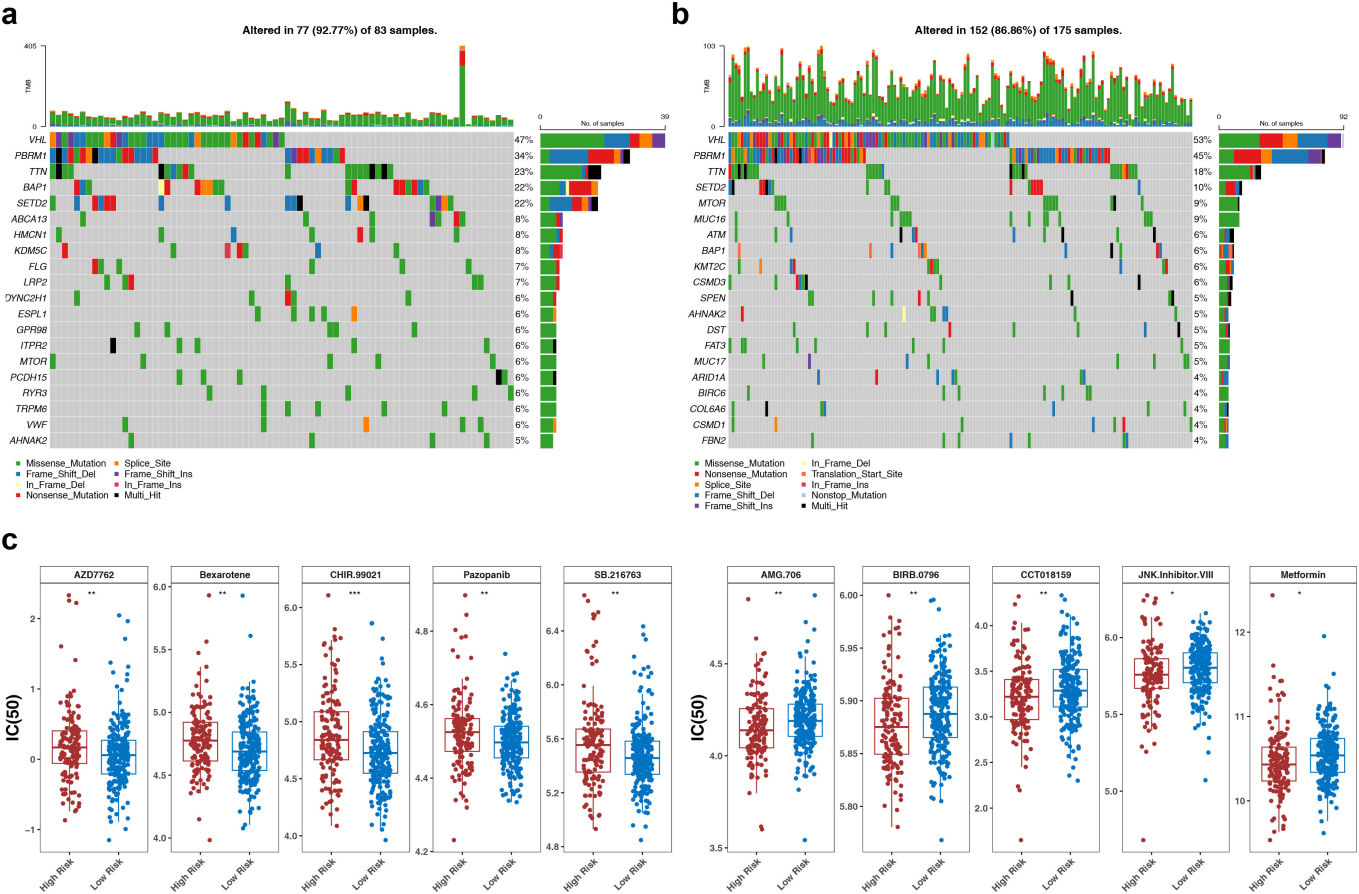
Somatic mutation analysis and drug sensitivity analysis. The top 20 mutated genes in the high-risk group **(a)** and the low-risk group **(b)**, with different colors representing different mutation types. **(c)** Drug sensitivity analysis plots of the high-risk group and the low-risk group. *p<0.05, **p<0.01, ***p<0.001.

In the results of the mutation analysis, the top 20 most frequently mutated genes in the HRG and in the LRG were presented. It was found that genes such as VHL and PBRM1 were significantly mutated in both the HRG (**Figure 7a**) and LRG (**Figure 7b**). In addition, the most frequent type of mutation among these genes was missense mutation. After that, in the analysis of chemotherapeutic drug sensitivity, 19 chemotherapeutic drugs showed significant differences in sensitivity between the HRG and LRG patients (p<0.05). The top 5 drugs such as CHIR.99021, Bexarotene with significant differences IC_50_ values were sorted in ascending order according to the p values (p < 0.05) (**Figure 7c**). The results showed that these drugs might have potential therapeutic effects on ccRCC.

### 3.8 Identification of MCs as key cells in ccRCC

**Figure 8:**
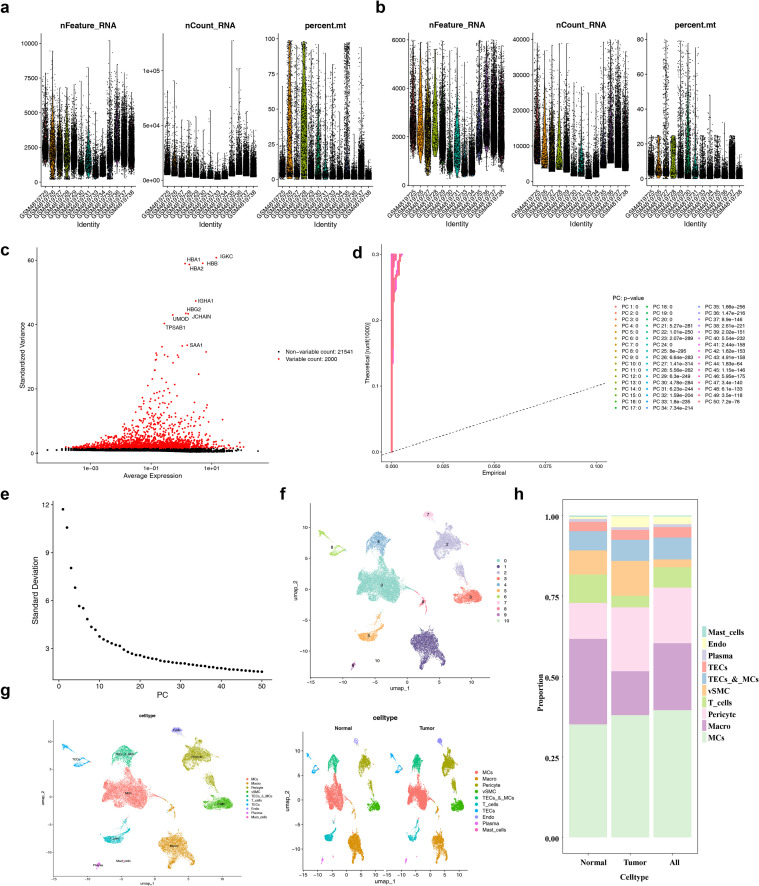
Single - cell analysis and cell clustering. Before **(a)** and after **(b)** quality control of single - cell data. **(c)** Screening of highly variable genes, with highly variable genes shown in red. **(d)** PCA dimensionality reduction plot. **(e)** PCA scree plot. **(f)** Cell UMAP dimensionality reduction and clustering plot. **(g)** Visualization of annotated cell clusters (ungrouped and grouped). **(h)** Proportion plots of cells in the Normal, Tumor, and All (Normal + Tumor) groups.

A total of 28,537 cells and 23,541 genes were obtained in the GSE159115 dataset (**Figure 8a**). After data quality control, the count of cells was 26,378, and the count of genes remained 23,541 (**Figure 8b**). Then, 2,000 HVGs were identified, with the top 10 HVGs highlighted (**Figure 8c**). Later, PCA was conducted, and it was found that the distribution between the samples and the groups was relatively uniform, and there was no batch effect (**Figure 8d**). And the standard deviation of the first 30 PCs changed significantly, while the change trend became smaller after the 30th PC, so the top 30 PCs were selected (p < 0.05) (**Figure 8e**). The UMAP analysis illustrated that the cells were clustered into 11 distinct clusters (**Figure 8f**). These clusters were then annotated as belonging to 10 cell clusters (**Figure 8g**). Further stacked bar chart analysis demonstrated that the proportions of MCs were different between the ccRCC and control samples, and accounted for the highest proportion in tumor samples (**Figure 8h**). Notably, RGS20, ADA, AICDA, and SLC12A5 were differentially expressed in MCs, and their expression levels were relatively high (**Supplementary Table 8**) (p < 0.05). Based on these findings, MCs were identified as key cells associated with ccRCC.

### 3.9 Exploring the cell enrichment pathways, interactions, and differentiation of MCs

**Figure 9:**
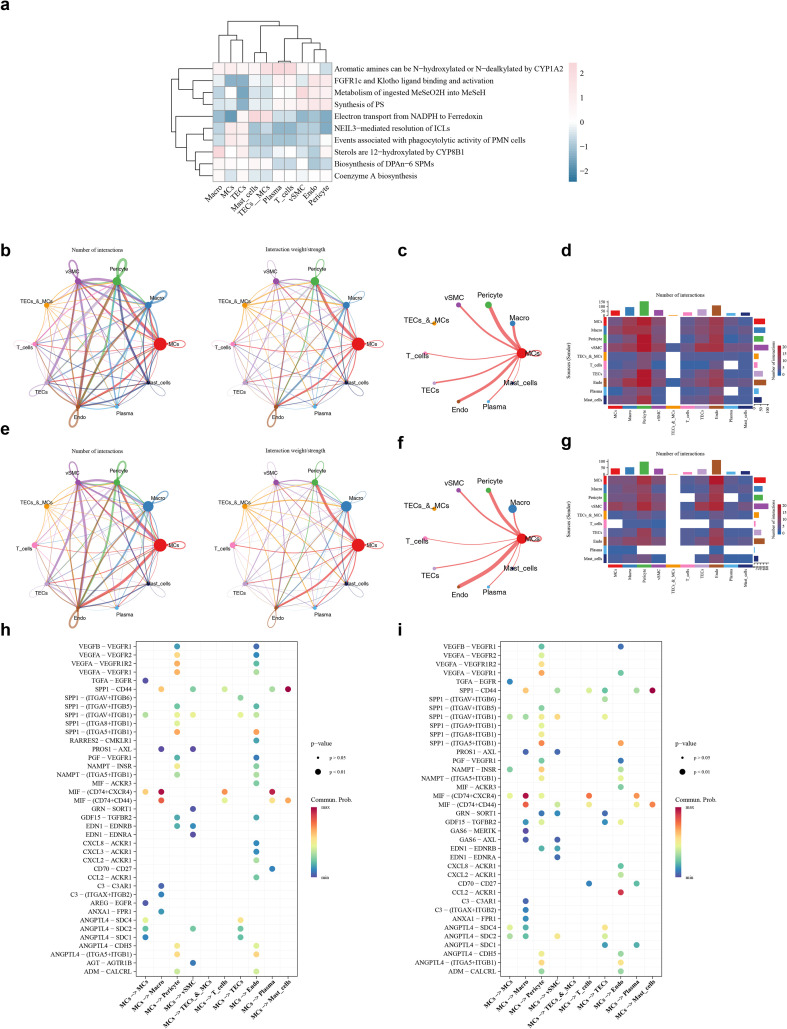
Subgroup cell pathway enrichment analysis and cell communication. **(a)** Differential pathway enrichment analysis, with colors ranging from light blue to pink representing the relative activity scores from low to high. **(b)** Quantity and intensity maps of the cell ligand - receptor interaction network in the Normal group. The size of the circles of various colors on the periphery indicates the number of cells. The larger the circle, the more cells or the higher the intensity. **(c)** Single - network diagram of the key cells MCs in the Normal group. **(d)** Heatmap of the number of interactions among key cells in the Normal group. **(e)** Quantity and intensity maps of the cell ligand - receptor interaction network in the Tumor group. **(f)** Single - network diagram of the key cells MCs in the Tumor group. **(g)** Heatmap of the number of interactions among key cells in the Tumor group. **(h)** Communication probability analysis of ligand - receptor between MCs and other cells in the Normal group and **(i)** in the Tumor group.

**Figure 10:**
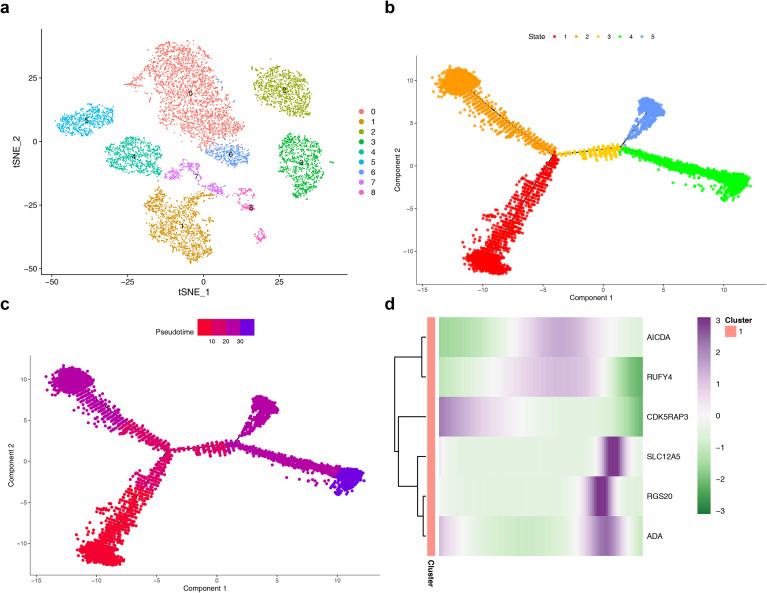
Pseudotemporal analysis **(a)** Cell clustering map of MCs. **(b)** Trajectory map of different cell subpopulations. **(c)** Pseudotemporal trajectory map. **(d)** Heatmap of the expression of prognostic genes at different developmental time points in MCs cells. Different colors in the heatmap represent different expression levels.

Cell enrichment analysis demonstrated that MCs were primarily associated with the “Electron transport from NADPH to Ferredoxin” (p < 0.05) (**Figure 9a**) (**Supplementary Table 9**). Cell-to-cell communication analysis emphasized frequent interactions between MCs and Pericytes in ccRCC (**Figure 9b**), the number of interactions between macrophages and MCs was increased (**Figure 9c**). At the same time, the number of communications between MCs and T cells increased (**Figure 9d**). The frequent interactions between MCs and Endothelials in control samples (**Figures 9e–g**), highlighting their potential roles in immune response and cellular signaling networks of the tumor microenvironment. Notably, compared with the control samples (**Figure 9h**), the receptor-ligand pair with a relatively high frequency of communication was CCL2 − ACKR1 of MCs and Endothelials in the ccRCC samples (**Figure 9i**), indicating potential differences in functions such as Inflammation and immunity, cell adhesion and migration. These findings demonstrated different signaling mechanisms of angiogenesis and cell migration in the context of the disease. Further analysis of macrophages through secondary dimensionality reduction and clustering revealed 9 distinct subclusters (**Figure 10a**). Pseudo-temporal analysis of macrophages contained 5 differentiation stages (**Figure 10b**), and differentiation uncovered a progressive trajectory from an early (light red) to a more mature (dark purple) state (**Figure 10c**). RGS20, SLC12A5, and ADA exhibited markedly higher expression in the middle-to-late differentiation stages; AICDA and RUFY4 demonstrated peak expression during the middle stage; CDK5RAP3 showed robust early-stage expression, followed by a gradual decline (**Figure 10d**). This indicated that 6 prognostic genes were involved in the expression of key cells.

## 4 Discussion

ccRCC remains a major clinical challenge due to its late presentation, high metastasis rate, and resistance to conventional therapies (33, 34). Ammonia-induced cell death (ammonitosis), a newly characterized regulated cell death pathway, has emerged as a critical player in cancer progression and immune modulation (7), but its role in ccRCC pathogenesis remains underexplored. In this study, we integrated bulk transcriptomics, scRNA seq, and experimental validation. Using differential analysis and machine learning algorithms, six differentially expressed genes (RGS20, ADA, AICDA, SLC12A5, RUFY4, CDK5RAP3) related to ammonia death were identified and obtained. MCs were identified as key cells, and the functional enrichment and immune infiltration of high-risk patients were explored. Meanwhile, the pseudo temporal analysis explored the dynamic expression of prognostic genes during key cell differentiation processes. This study systematically analyzed the role of ADRGs in ccRCC for the first time and found that six prognostic genes were significantly upregulated in ccRCC and associated with reduced patient survival. This discovery suggests a potential link between ammonia metabolism-related genes and disease progression in ccRCC, warranting further investigation into its clinical relevance.

Regulator of G protein signaling 20 (RGS20) is a negative regulator of GPCR signaling, but its role in cancer is context-dependent (35). In ccRCC, we found that RGS20 is highly expressed in tumor tissues and correlates with advanced stage and poor survival. This is consistent with a recent study showing that RGS20 promotes ccRCC cell migration and invasion by activating the PI3K/AKT pathway (36). GPCRs are major drivers of ccRCC progression, for example, the von Hippel-Lindau (VHL) tumor suppressor gene, frequently mutated in ccRCC, regulates GPCR-mediated signaling (37). Our data suggest that RGS20 may enhance GPCR signaling in ccRCC by modulating downstream effectors, thereby promoting tumor growth.

Adenosine deaminase (ADA) and activation-induced cytidine deaminase (AICDA) are key regulators of immune function. ADA catalyzes the conversion of adenosine to inosine, a process that suppresses T cell activation by reducing extracellular adenosine levels (38). Research has shown that the decrease in ADA activity is closely related to disease progression in cancers such as head and neck cancer, prostate cancer, and laryngeal squamous cell carcinoma (38). In ccRCC, we found that ADA expression is positively correlated with immune checkpoint molecules (e.g., PD-L1) and regulatory T cell (Treg) infiltration. This suggests that ADA may promote immune evasion by creating an immunosuppressive TME.

AICDA (Activation-Induced Cytokine Deaminase) is a core regulatory enzyme of adaptive immunity, mainly expressed in activated B cells (germinal center B cells) and present in small amounts in T cells and other immune cells, mediating class switching recombination (39). However, recent work has identified AICDA in tumor cells, where it promotes genetic instability and therapy resistance (40). In addition, studies have found that AICDA is an oncogene that may induce tumorigenesis through DNA demethylation, and targeting this gene may represent a novel therapeutic approach for metastatic ccRCC (41). In our study, AICDA was highly expressed in ccRCC and correlated with B cell infiltration. Given that B cells in the TME can either promote (e.g., regulatory B cells) or inhibit (e.g., effector B cells) tumor growth, future studies should clarify whether AICDA modulates B cell function in ccRCC.

Solute carrier family 12 member 5 (SLC12A5) is a potassium chloride co transporter protein primarily involved in maintaining intracellular ion homeostasis. Research has shown that SLC12A5 has carcinogenic effects in various cancers, and its overexpression is associated with tumor progression, invasion, metastasis, and poor prognosis. For example, SLC12A5 inhibits apoptosis by suppressing apoptosis inducing factor (AIF) and Endonuclease G(EndoG)-dependent apoptosis signaling pathways, thereby promoting tumor cell survival. Additionally, it can promote tumor cell invasion and metastasis by regulating matrix components such as Matrix Metalloproteinases (MMPs) and Fibronectin (FN) (42). In prostate cancer, SLC12A5 may promote tumor progression and drug resistance by interacting with m6A reader YTHDC1 and transcription factor HOXB13 (43). The above evidence is consistent with our research conclusion that high SLC12A5 expression is associated with poor survival rate.

RUFY4 (RUN and FYVE domain-containing 4) regulates cytoskeleton dynamics by interacting with Rab GTPases (44), while CDK5RAP3 (CDK5 regulatory subunit-associated protein 3) modulates cell cycle progression via CDK5 inhibition (45). In addition, RUFY4 positively correlates with the expression of programmed death-ligand 1 (PD-L1), and the absence of RUFY4 leads to a decrease in PD-L1 expression, indicating that RUFY4 plays a significant role in immune regulation and immunotherapy (46). In ccRCC, we found that both genes are upregulated in tumor tissues and correlate with increased cell proliferation (e.g., Ki-67 expression). These results indicate that RUFY4 and CDK5RAP3 are associated with poor survival in patients.

Collectively, these data suggest that the 6 ADRGs promote ccRCC through a network of metabolic, immune, and proliferative pathways, highlighting the complexity of ammonia toxicity mediated tumor progression. The scRNA-seq revealed that MCs are the dominant cell type in ccRCC and the primary expressers of the 6 prognostic genes. This is consistent with previous scRNA-seq studies showing that MCs drive tumor progression by secreting growth factors (e.g., VEGF) and modulating the TME (49). MCs refer to the basic building blocks of cancer that break through normal growth regulation mechanisms due to genetic mutations or epigenetic changes. MCs can experience immune escape, leading to uncontrolled proliferation and enhanced invasion and metastasis capabilities (47). Research has shown that the MCs of ccRCC are prone to invade the renal vein and form cancer thrombi, which can later metastasize to organs such as the lungs, bones, and liver (26, 48). Our intercellular communication analysis further confirmed that MCs interact with T cells and macrophages via ligand-receptor pairs such as CXCL12-CXCR4 and IL-6-IL6R. These interactions are known to suppress anti-tumor immunity: CXCL12-CXCR4 recruits Tregs and myeloid-derived suppressor cells (MDSCs), while IL-6-IL6R promotes M2 macrophage polarization (49).

Through pseudo-time series analysis, it was found that the expression of the 6 prognostic genes increases as MCs differentiate from a “quiescent” to a “proliferative” state, suggesting that these genes are involved in tumor progression and therapy resistance. For example, RGS20 and CDK5RAP3 expression was highest in late-stage MCs, correlating with increased cell cycle activity (45, 50). This aligns with studies showing that tumor cell differentiation status is a key predictor of ccRCC survival.

Notably, MCs from high-risk patients had higher expression of immune checkpoint molecules (e.g., PD-L1) and chemokines (e.g., CCL2) compared to those from low-risk patients. This suggests that the prognostic model not only predicts survival but also reflects the immunogenicity of the tumor—an important consideration for immunotherapy selection.

While our study provides valuable insights into ccRCC pathogenesis, several limitations warrant consideration. First, the bulk transcriptomic data were derived from TCGA and ICGC, which may have geographic biases and limited clinical information (e.g., treatment history). Second, the scRNA-seq cohort was small (7 ccRCC patients, 6 normal controls), limiting the generalizability of our cell-type-specific findings. Third, we only validated gene expression using RT-qPCR; functional studies (e.g., knockdown/overexpression in cell lines, animal models) are needed to confirm the causal roles of the 6 ADRGs in ccRCC. Fourth, the mechanism by which these genes regulate ammonitosis (e.g., ammonia production, lysosomal stability) remains unclear and requires further investigation.

Future studies should address these limitations by: (1) Validating the prognostic model in multi-center cohorts with detailed clinical data; (2) Expanding scRNA-seq analysis to larger cohorts to characterize MC heterogeneity; (3) Performing functional experiments to elucidate the mechanistic links between ADRGs, ammonitosis, and ccRCC progression; (4) Exploring combination therapies targeting the 6 ADRGs and immune checkpoints (e.g., anti-PD-L1 + anti-ADA); (5) Developing circulating biomarkers (e.g., plasma ADRG mRNA) for non-invasive prognosis.

## 5 Conclusion

In summary, our study screened out six ADRGs in ccRCCC and constructed a risk model based on these six genes. The risk model shows promise as a potential adjunct tool for risk stratification in ccRCC, and the mechanistic insights into ammonia metabolism suggest novel avenues for therapeutic exploration. However, further validation in independent cohorts and comparison with established prognostic models are needed before clinical translation. These findings bridge the gap between basic science and clinical oncology, paving the way for more personalized and effective treatments for ccRCC patients.

## Data Availability

The datasets presented in this study can be found in online repositories. The names of the repository/repositories and accession number(s) can be found in the article/**Supplementary Material**.

## Supplementary material

Supplementary Table 1 ADRGs information.

Supplementary Table 2 Primer sequence table.

Supplementary Table 3 GO enrichment analysis pathway results.

Supplementary Table 4 KEGG enrichment analysis pathway results.

Supplementary Table 5 Schoenfeld test scatter plot of candidate prospective genes.

Supplementary Table 6 Enrichment analysis of GSEA pathway in HRG and LRG patients.

Supplementary Table 7 Enrichment analysis of GSVA pathway in HRG and LRG patients.

Supplementary Table 8 Box plot of differential analysis of prognostic genes in cells, where ns represents insignificant, *p < 0.05, **p < 0.01, ***p < 0.001, ****p < 0.0001.

Supplementary Table 9 Cell enrichment analysis.
