# Supplementary figures and images for: Identification of a prognostic signature based on ammonia metabolism-related genes in clear cell renal cell carcinoma: an integrated analysis of bulk and single-cell transcriptomics
Source: Front Immunol. 2026 Apr 23;17:1765098. doi: 10.3389/fimmu.2026.1765098 (PMC13149443; doi:10.3389/fimmu.2026.1765098)

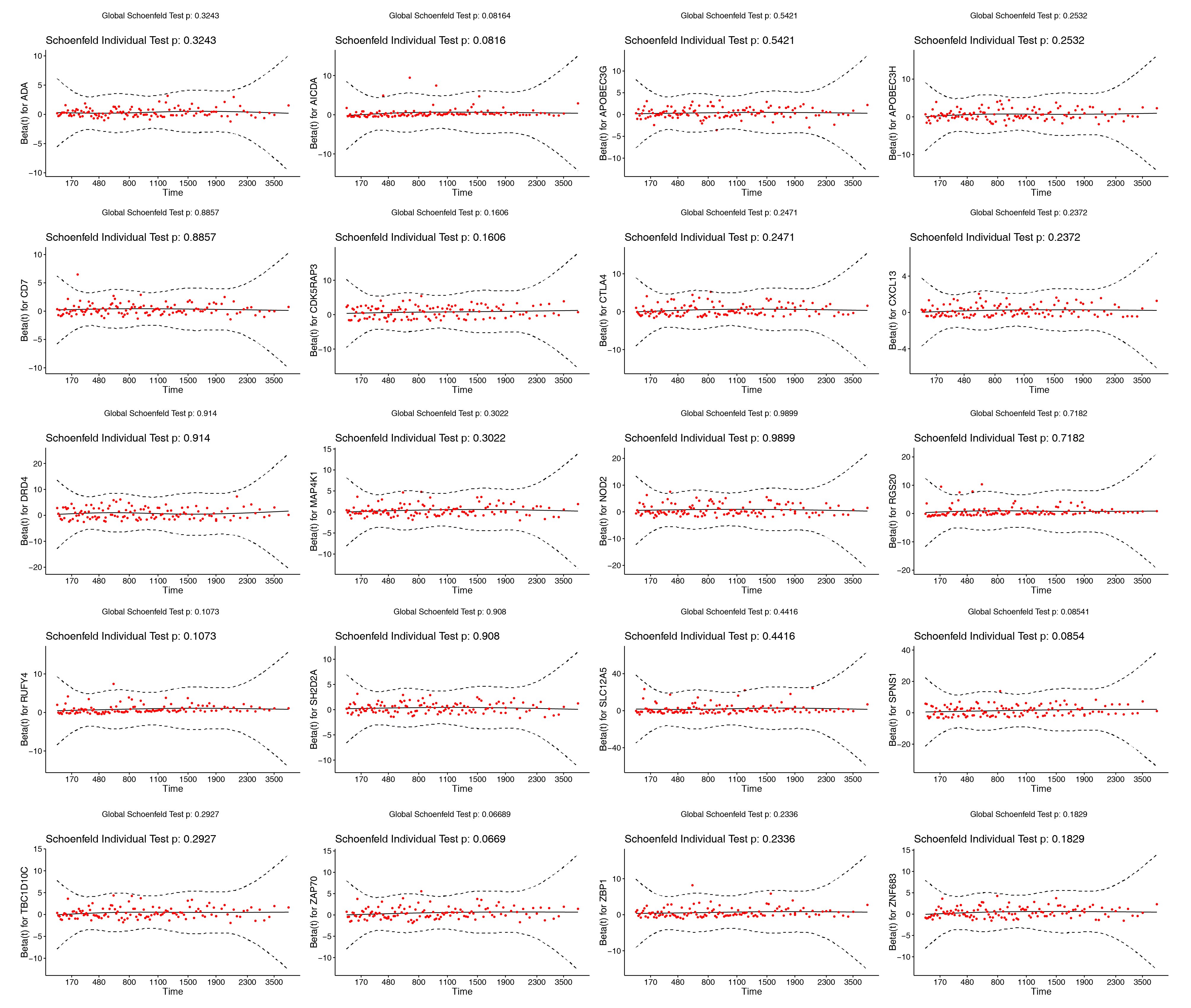

Supplement: Supplementary Table 5 — Schoenfeld test scatter plot of candidate prospective genes. [file Supplementaryfile5.tif]

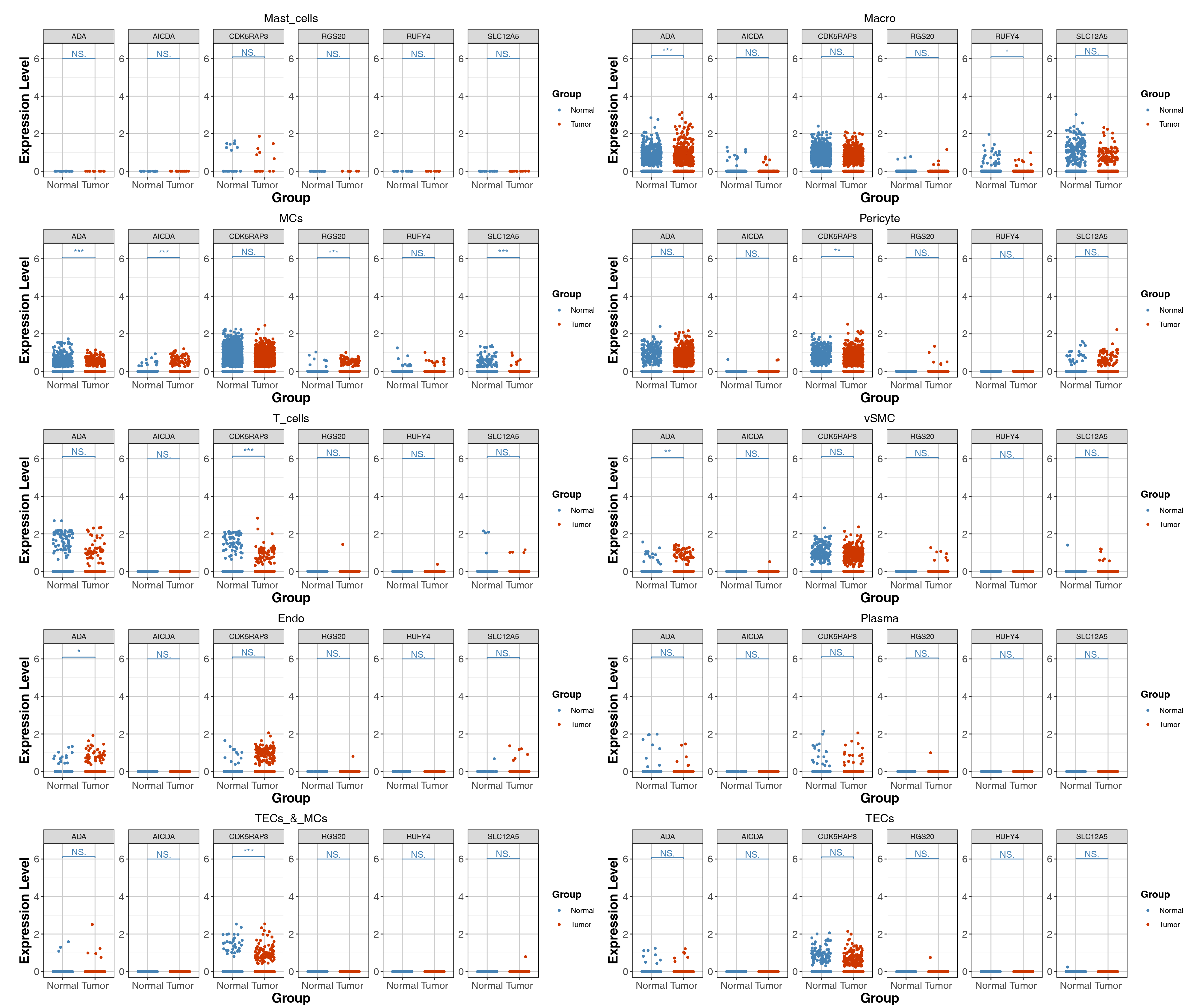

Supplement: Supplementary Table 8 — Box plot of differential analysis of prognostic genes in cells, where ns represents insignificant, *p < 0.05, **p < 0.01, ***p < 0.001, ****p < 0.0001. [file Supplementaryfile8.tif]
